# Primary Mediastinal Large B-Cell Lymphoma during Pregnancy

**DOI:** 10.1155/2012/197347

**Published:** 2012-10-31

**Authors:** Cesar A. Perez, Janki Amin, Luz M. Aguina, Maureen Cioffi-Lavina, Edgardo S. Santos

**Affiliations:** ^1^Division of Hematology/Oncology, Sylvester Comprehensive Cancer Center, University of Miami Miller School of Medicine, 1475 NW 12th Avenue, Miami, FL 33136, USA; ^2^Department of Anesthesiology, Perioperative Medicine and Pain Management, University of Miami Miller School of Medicine, 1475 NW 12th Avenue, Miami, FL 33136, USA; ^3^Department of Pathology, University of Miami Miller School of Medicine, 1475 NW 12th Avenue, Miami, FL 33136, USA

## Abstract

Non-Hodgkin's Lymphoma (NHL) rarely presents during pregnancy and primary mediastinal large B-cell lymphoma (PMLBCL) accounts for approximately 2.5% of patients with NHL. The case of a 22-year-old woman who was diagnosed with Stage IIA PMLBCL during week 13 of her intrauterine pregnancy is described. The staging consisted in computed tomography (CT) of the chest and magnetic resonance imaging (MRI) of the abdomen and pelvis. She was managed with R-CHOP regimen (rituximab, cyclophosphamide, doxorubicin, vincristine, and prednisone) for a total of six cycles and, because of the early presentation during the second trimester, she received the entire chemotherapy course during the pregnancy. She delivered a healthy baby at 34 weeks of pregnancy and a ^18^FDG-PET/CT scan demonstrated complete remission after delivery. After 20 months of follow up she remains with no evidence of disease and her 1-year-old son has shown no developmental delays or physical abnormalities. PMLBCL, although an uncommon subgroup of DLBCL, may present during pregnancy and R-CHOP should be considered as one suitable option in this complex scenario.

## 1. Introduction

Hodgkin's lymphoma (HL) is the most frequent hematologic malignancy in pregnant women, with an incidence rate ranging from 1 to 1000–6000. In contrast, non-Hodgkin's Lymphoma (NHL) uncommonly presents during pregnancy, with estimated incidence of 0.8 cases per 100,000 women [1, 2]. This low incidence when compared to the age-adjusted general female population may be explained by the fact that hormone therapy is associated with reduced risk of NHL, suggesting that exposure to reproductive hormones during pregnancy may promote favorable immunologic responses that decrease the risk of developing NHL [3]. Lymphoma during pregnancy usually presents at a median of 24 weeks of gestation and have been reported to have a good outcome with minimal maternal and fetal complications [4]. Primary mediastinal large B-cell lymphoma (PMLBCL) is a variant of diffuse large B-cell lymphoma (DLBCL), and it accounts for approximately 2.5% of the patients with NHL, with a specific predilection for young female adults [5]. The diagnosis and management of hematologic malignancies during pregnancy brings to light complex ethical issues and often requires a multidisciplinary team to undertake the challenge of optimizing the outcome of both mother and fetus. We report a case of a 22-year-old woman diagnosed with PMLBCL early in her second trimester of pregnancy and the specific complexities of the management of this never reported scenario. 

## 2. Case Report

**Figure 1:**
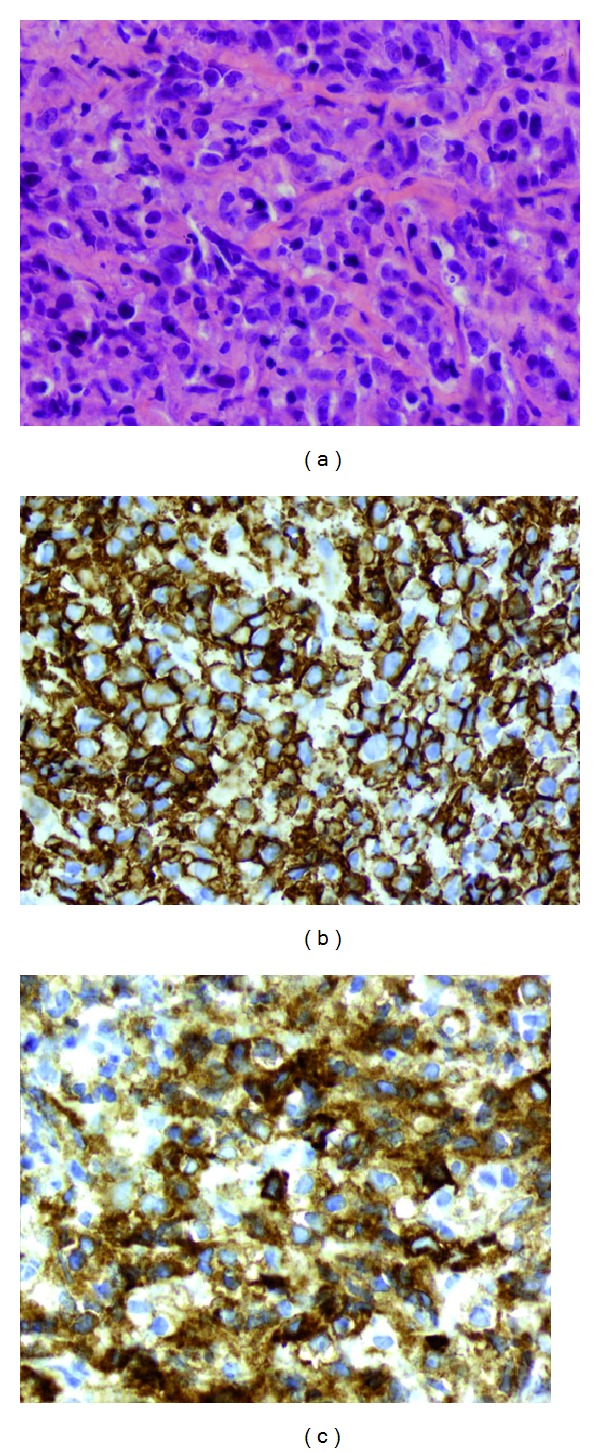
Primary mediastinal large B-cell lymphoma. (a) Hematoxylin and eosin (50X). Primary mediastinal B cells (PMBC) associated with delicate interstitial fibrosis. (b) PMBC immunoreactivity for B cell antigen on the membrane, CD20. (c) PMBC immunoreactivity for CD30.

The patient is a 22-year-old woman who presented during the week 12 of her intrauterine pregnancy with one-month history of palpitations and intermittent chest pain, but no B-symptoms. The only abnormality on physical exam was a grade III/VI systolic murmur noted in the left upper sternal border. An echocardiogram revealed right ventricular systolic pressure of 60 mmHg, but normal left-ventricular ejection fraction. Blood tests revealed normal blood counts and chemistry, but an elevated lactate dehydrogenase at 1627 IU/L. An initial chest X-ray and follow-up computed tomography (CT) of the chest revealed a large mass in the mediastinum measuring 11 × 13 cm extending into the left neck and compressing the main pulmonary artery. She thereafter underwent a CT-guided biopsy of the mediastinal mass that revealed large-sized cells associated with compartmentalizing alveolar fibrosis (Figure 1(a)) that were positive for CD20, CD30 (Figures 1(b) and 1(c)), and CD45 and negative for CD3, CD10, CD15, CD34, TdT, CD23, MUM-1, and keratin. A diagnosis of PMLBCL was established. A bone marrow biopsy showed no immunophenotypic evidence of malignant involvement. Because of her pregnancy, her abdominal staging was done with a magnetic resonance imaging (MRI) of the abdomen and pelvis that found no abnormalities. She was staged as stage IIA PMLBCL with bulky disease in the mediastinum.

After a multidisciplinary consensus, the patient was started on treatment with standard doses of R-CHOP regimen (rituximab, cyclophosphamide, doxorubicin, vincristine, and prednisone) given every 3 weeks, for a total of six cycles. Her treatment started during the 13th week of pregnancy and her last chemotherapy was given at her 31st week of pregnancy. A fetal echocardiogram was done at week 28th of pregnancy and revealed no evidence of structural or functional heart disease on the fetus. She completed treatment with no complications but fatigue grade 1 and received dexamethasone for fetal lung maturation since the prednisone given in the R-CHOP regimen is known not to cross the blood-placental barrier. At week 34 4/7 of pregnancy the patient underwent induction of labor, delivering a healthy baby with Apgar score of 9/9. A positron emission tomography/computed tomography scan was done one week after delivery and revealed complete resolution of the mediastinal mass. She then underwent consolidation radiation therapy to the mediastinum for a total of 36 Gy in 20 fractions. After 20 months of follow up she remains with no evidence of disease and her 1-year-old son has shown no developmental delays or physical abnormalities.

## 3. Discussion

PMLBCL was classified as a distinct subtype of DLBCL initially by the Revised European and American Classification of Lymphoid neoplasms and in 2001 by the World Health Organization [6, 7]. Epidemiologically, PMLBCL is more common in women than men and has a median age of 37 at presentation [5]. Conversely, DLBCL often presents in the elderly population and commonly in men, making PMLBCL an entity relevant for the pregnant population and possibly an underreported scenario because of its relatively recent introduction into the lymphoma classification [8]. 

The disease often presents as a bulky tumor in the mediastinum, causing compressive symptoms including dyspnea and superior vena cava syndrome. Pleural or pericardial effusions have been found at presentation in up to 50% of patients. While bone marrow infiltration is rare, extranodal sites may be involved in recurrent disease [8]. PMLBCL arises from a population of thymic B cells and consists of medium-sized to large cells with an abundant pale cytoplasm and they are commonly associated with a delicate interstitial fibrosis. Immunohistochemistry of the cells shows the presence of B-cell antigens, including CD19, CD20, CD22, and CD79a, in addition to CD30 positivity in more than 80% of cases [8]. While genomic profiling has allowed PMLBCL to be distinguished from DLBCL, it has also demonstrated similarities between the transcription profile of PMLBCL and Hodgkin lymphoma. Specifically, over one-third of all PMLBCL signature genes, including MAL, SNFT, TNFRSF6, TARC, and CD30, were found to be highly expressed in the Hodgkin lymphoma [9]. 

The management of PMLBCL has been a longstanding matter of discussion. CHOP regimen has yielded poor results, with cure rates rarely surpassing 50–60% [5]. Studies with more aggressive regimens as MACOP-B (methotrexate, cytarabine, cyclophosphamide, vincristine, prednisone, and bleomycin) followed by involved field radiation therapy demonstrated better results, with complete remission rates of 86% and overall survival (OS) and progression-free survival (PFS) rates of 86% and 91%, respectively, at nine years [10]. However, the use of methotrexate during pregnancy should be discouraged because of the known teratogenic effects of the agent even in advanced stages of the pregnancy [11]. One of the most promising approaches to PMLBCL has been the use of EPOCH regimen (etoposide, vincristine, doxorubicin, cyclophosphamide, and prednisone) with and without rituximab. Dunleavy et al. reported an OS of 100% and event-free survival (EFS) of 94% with EPOCH plus rituximab in 22 patients with PMLBCL [12]. Unfortunately, the lack of evidence of etoposide in pregnancy limits the feasibility of this regimen in pregnant patients. Recently, R-CHOP regimen was used in 76 patients with PMLBCL and demonstrated to be superior to CHOP alone and comparable to more intensive chemotherapy regimens, with cure rates exceeding 82% and 5-year OS rates of 89% [5]. In addition, a review of NHL treated during pregnancy with CHOP demonstrated that patients could be offered standard regimens with no evidence of adverse fetal events. Likewise, the addition of rituximab was not associated with higher incidence of fetal malformations [13]. 

## 4. Conclusions

The specific predilection of PMLBCL for young female adults makes it an important entity to be consider among of the hematological malignancies during pregnancy. R-CHOP is a suitable option in this complex scenario since good outcomes for both mother and fetus can be achieved even when the therapy is delivered entirely during the pregnancy.
